# Utility of Intraoperative Neuromonitoring during Minimally Invasive Fusion of the Sacroiliac Joint

**DOI:** 10.1155/2014/154041

**Published:** 2014-12-04

**Authors:** Michael Woods, Denise Birkholz, Regina MacBarb, Robyn Capobianco, Adam Woods

**Affiliations:** ^1^Missoula Bone and Joint, 2360 Mullan Road, Suite C, Missoula, MT 59808, USA; ^2^Pronerve, 7600 East Orchard Road, Suite 200, Greenwood Village, CO 80111, USA; ^3^SI-BONE, Inc., 3055 Olin Avenue, Suite 2200, San Jose, CA 95128, USA

## Abstract

*Study Design*. Retrospective case series. *Objective*. To document the clinical utility of intraoperative neuromonitoring during minimally invasive surgical sacroiliac joint fusion for patients diagnosed with sacroiliac joint dysfunction (as a direct result of sacroiliac joint disruptions or degenerative sacroiliitis) and determine stimulated electromyography thresholds reflective of favorable implant position. *Summary of Background Data*. Intraoperative neuromonitoring is a well-accepted adjunct to minimally invasive pedicle screw placement. The utility of intraoperative neuromonitoring during minimally invasive surgical sacroiliac joint fusion using a series of triangular, titanium porous plasma coated implants has not been evaluated. *Methods*. A medical chart review of consecutive patients treated with minimally invasive surgical sacroiliac joint fusion was undertaken at a single center. Baseline patient demographics and medical history, intraoperative electromyography thresholds, and perioperative adverse events were collected after obtaining IRB approval. *Results*. 111 implants were placed in 37 patients. Sensitivity of EMG was 80% and specificity was 97%. Intraoperative neuromonitoring potentially avoided neurologic sequelae as a result of improper positioning in 7% of implants. *Conclusions*. The results of this study suggest that intraoperative neuromonitoring may be a useful adjunct to minimally invasive surgical sacroiliac joint fusion in avoiding nerve injury during implant placement.

## 1. Introduction

Minimally invasive (MIS) sacroiliac (SI) joint fusion has gained popularity as a safe and effective treatment option for patients with recalcitrant symptoms of SI joint degeneration or disruption (based on joint asymmetry via radiographic imaging or contrast leakage during diagnostic joint block) [1, 2]. A common method uses a series of triangular, titanium porous plasma spray (TPS) coated implants (iFuse Implant System, SI-BONE, Inc; San Jose, CA) [3].

**Figure 1:**
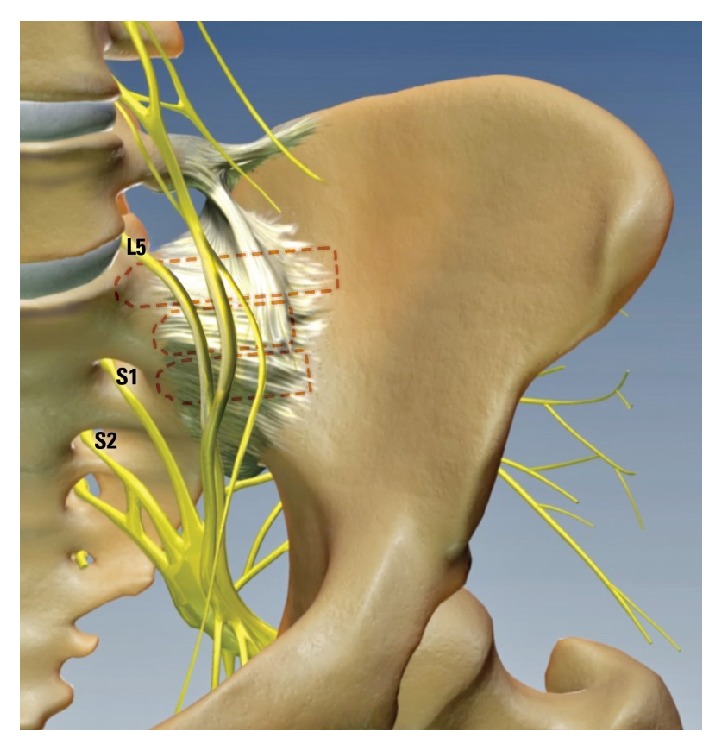
A three-dimensional representation of the lumbar spine, sacrum, and ilium. Red dashed outlines depict common positioning of three iFuse implants across the sacroiliac joint. Of the neural structures shown, the L5, S1, and S2 nerves (labeled in the figure) are at greatest risk of injury during such procedures.

Similar to MIS pedicle screw procedures, surgery is performed under indirect visualization using fluoroscopic guidance and the implants are placed in bone adjacent to several neural structures. Achieving clear visualization can be difficult as the trajectory is much more anatomically complex than in lumbar spinal procedures (Figure 1). Bony landmarks are often obscured and can therefore be misinterpreted [4]. Thus, there is a potential risk for neural encroachment due to improper implant placement with possible neurologic sequelae. Given the consequences of iatrogenic nerve injury, it is advisable to employ neurologic structure localization techniques.

Intraoperative neuromonitoring (IOM) is a well-documented technique used to decrease the risk of iatrogenic nerve injury during MIS spinal procedures performed under limited visualization [5, 6]. This technology is dependent upon the greater electrical resistance of bone compared to the surrounding fluid and soft tissue. As a result, an implant that is entirely embedded in bone will be electrically shielded within certain limits from adjacent neural structures [7]. Should the implant come within close proximity to a nerve, the implant will no longer be electrically shielded, resulting in neural stimulation upon passage of current through the implant. The resultant response of the muscle that is being monitored alerts the surgeon to possible implant misplacement so that action may be taken to avoid potential neurologic sequelae.

Several methods of neuromonitoring have been utilized in spinal procedures, including electromyography (EMG) recordings, somatosensory evoked potentials, and motor-evoked potentials [8–10]. In comparison to EMG monitoring, the latter methods monitor the ascending and descending pathways of the spinal cord, which are not considered to be at risk during SI joint fusion surgery. Conversely, intraoperative EMG monitoring provides the surgeon with real-time neurophysiological feedback from the individual nerve roots at risk, enabling a rapid response if unfavorable readings are obtained. Intraoperative EMG has a high documented success rate for detecting the proximity of an implant or instrument to neural structures during spinal procedures [7, 11, 12]. Given the potential benefits of EMG monitoring, the purpose of the present study is to document its clinical utility during MIS SI joint fusion and determine stimulated EMG thresholds reflective of favorable implant position.

## 2. Materials and Methods

After IRB approval was obtained, a medical chart review was undertaken for consecutive patients operated on by a single orthopedic surgeon (MW). Thirty-seven consecutive patients, treated with MIS SI joint fusion using IOM between November 2012 and January 2014, were identified. Data extracted from the medical chart included demographics, comorbidities, neurological status, pertinent medical history related to the lumbopelvic hip complex, relevant imaging studies, estimated blood loss, and perioperative and postoperative complications. Stimulated EMG thresholds during surgery were obtained directly from the neuromonitoring technologist's records.

The primary endpoint of this study was the relationship of EMG threshold values to device reposition and overall success of the surgery defined by lack of reoperation within 30 days and absence of postoperative neurological impairment.

### 2.1. Procedure Description

**Figure 2:**
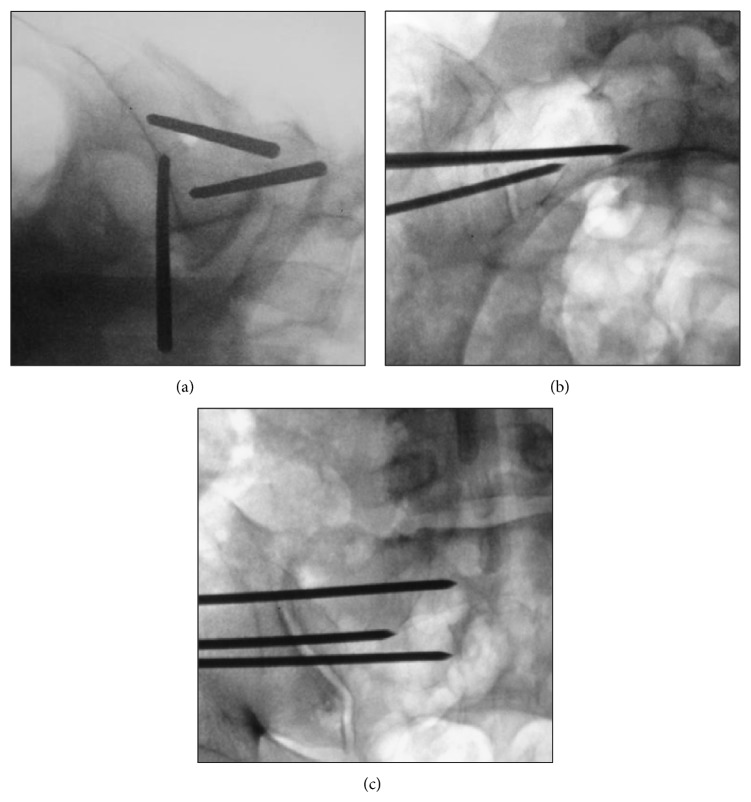
Lateral (a), inlet (b), and outlet (c) views of guide wire placement through the ilium, across the sacroiliac joint, and into the sacrum. Note the difficulty in visualizing the sacral foramen in (c).

**Figure 3:**
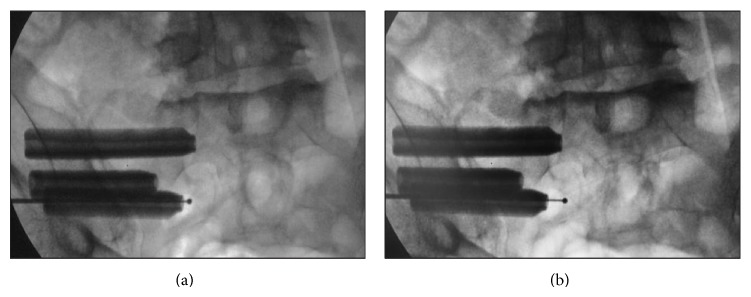
Following implant placement, the neuromonitoring probe is placed through the cannula of the implant and set to “searching” mode at 8 mA. In (a), monitoring of the probe past the distal end of the implant reveals a safe response, while further advancement of the probe to the position shown in (b) results in a positive EMG response, indicating that the probe has come within close proximity to a neural structure.

**Figure 4:**
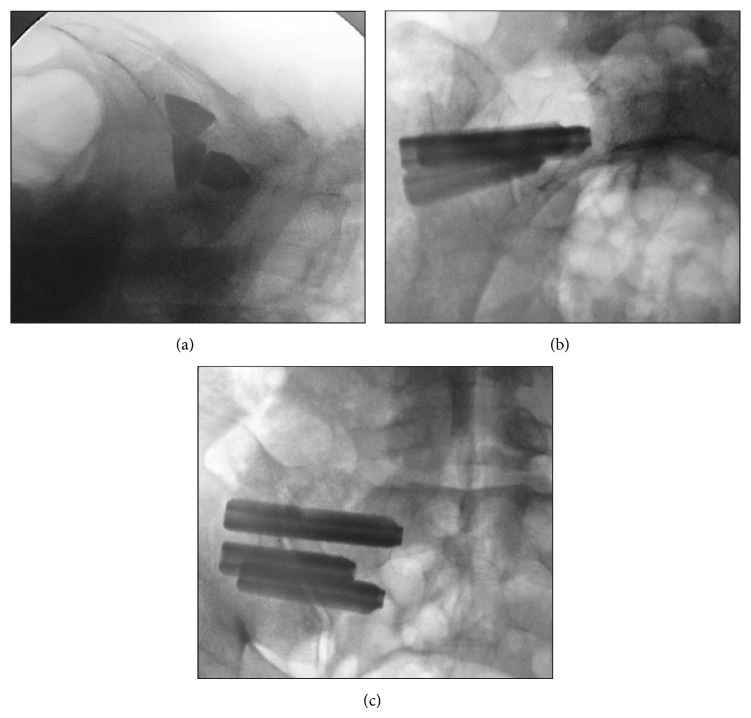
Lateral (a), inlet (b), and outlet (c) views of final implant placement through the ilium, across the sacroiliac joint, and into the sacrum, representing safe implant placement as measured via continuous passive EMG monitoring and fluoroscopy.

Prior to surgery, a computed tomography (CT) scan was obtained to determine the presence of anomalous sacral anatomy. MIS SI joint fusion surgery using a series of triangular, titanium porous plasma (TPS) coated titanium implants was performed with the patient on a radiolucent table to facilitate the use of intraoperative fluoroscopy. After general endotracheal anesthesia was administered, the patient was connected via subdermal needle electrodes to a standard neuromonitoring system (NIM-ECLIPSE, Medtronic, Memphis, TN). Continuous free-running EMG monitoring of selected muscle groups innervated by nerves at risk was employed throughout the surgery (IOM procedure description below). The patient was then carefully positioned prone, padded appropriately, and prepped in the normal sterile fashion. A lateral incision (3 cm) was made parallel to the sacral body as viewed on a lateral fluoroscopic image. The gluteal fascia was then incised in line with the incision and the gluteal musculature was bluntly dissected to reach the outer table of the ilium. A 3.2 mm guide pin was passed through the ilium, across the SI joint, and into the sacrum lateral to the neural foramen using an insulated soft tissue retractor. The pin was intermittently stimulated at 8 milliamperes (mA) during advancement to ensure that no neurologic structures were encountered. After the first pin was in an acceptable position, two additional pins were sequentially placed caudally in a triangular pattern to reflect the anatomy of the joint and ensure optimal bony purchase (Figure 2). Pin length was measured to determine implant length. A larger soft tissue protector was then passed over the first pin and a center channel was drilled from the ilium, across the SI joint, and into the sacrum. Drill progression was monitored under fluoroscopy to prevent medial migration of the pin. A cannulated broach was used to prepare a triangular channel and the implant was then delivered into its final position. The guide pin was then removed. A standard ball-tip probe was passed through the center channel of the implant and was progressively stimulated up to a maximum of 20 mA. The implant was considered to be in a safe position if no distal muscle activity was detected at or below 16 mA. If activity was detected, a “search” technique was used, where the probe was set to a constant current of 8 mA and slowly advanced out of the distal end of the implant under fluoroscopic guidance in an effort to identify the location of the adjacent neurologic structure (Figure 3). Based on this information, the implant position was modified if necessary, typically by retracting its position a few millimeters laterally. If the implant was adjusted, stimulation was repeated (as previously described) to ensure proper positioning. This technique was repeated for the remaining 2 guide pins; all 37 patients received 3 implants (Figure 4). Following placement of all implants, the wound was irrigated, final hemostasis was achieved, tissue layers were closed, and local anesthetic was injected. A postoperative CT scan to evaluate final implant position was obtained if clinically indicated but was not performed routinely to avoid excessive patient radiation exposure.

A program of gradual return to full weight bearing was prescribed for all patients. In general, patients were instructed to ambulate 50% weight bearing with the assistance of crutches or walker for the first 3 weeks; after which time a regimen of gradual return to full weight bearing was recommended. Postoperative physical therapy was tailored to individualized clinical need. Patients were followed up in the office at 2, 6, and 12 weeks postoperatively.

### 2.2. Intraoperative Neuromonitoring Protocol

Both free-running and stimulated-EMG are used during the procedure to detect any nerve irritation. Subdermal needles are placed to monitor muscle activity triggered by the corresponding nerve roots as follows: tibialis anterior for L4-5, extensor hallucis longus for L5-S1, and gastrocnemius, abductor hallucis brevis, and flexor digitorum brevis for S1-2. Once all 3 guide pins are placed, a current of 8 mA is applied to each pin, utilizing an insulated pin guide to prevent shunting. Lack of response at this threshold is used as an additional guideline to determine correct depth of penetration, in addition to fluoroscopic imaging. Once the first implant is sited and the guide pin is removed, a ball tip probe is passed into the cannulated implant. Current is gradually applied until a response is noted or the current reaches 20 mA. Any response noted at 16 mA or lower warrants close implant reevaluation. To assess implant proximity to the adjacent nerve structure(s), a “search” technique is also used. The ball tip probe is inserted through the cannulated implant and advanced past the distal end using a constant current of 8 mA (Figure 3). The probe is slowly advanced under fluoroscopic guidance until a response is noted.

## 3. Results

A total of 111 implants were placed in 37 patients. Eight (8/111) implants were repositioned in response to EMG thresholds of ≤16 mA.

Two false negative results were noted. One patient had an immediate repositioning of an implant based on a search mode reading that suggested close neural proximity. The second patient recorded a final EMG threshold of 17 mA, with good implant placement noted on intraoperative fluoroscopy. The patient reported new neurologic symptoms postoperatively. A CT scan done immediately postoperatively revealed the caudal implant in close proximity to, but not impinging on, a nerve root. The patient elected not to reposition immediately, but, after thirty days, the patient was returned to the operating room to revise the implant. Postoperatively, the patient's symptoms were somewhat improved but not resolved. Further evaluation revealed additional pathology unrelated to the SI joint (lumbar spinal stenosis and piriformis syndrome) requiring surgical intervention.

Three false positive results were obtained in one patient. Readings of 10 mA were obtained for all three implants in final position. Using intraoperative imaging and search mode techniques, all three implants were determined to be in an acceptable position. This was confirmed on postoperative CT imaging. Postoperatively, the patient experienced satisfactory relief of SI joint symptoms and no postoperative issues. The relationship between anatomy and the potential for false positives is discussed below.

EMG readings obtained for 111 implants resulted in 8 true positives, 3 false positives, 2 false negatives, and 98 true negatives. These results provide sensitivity and specificity rates of 80% and 97%, respectively.

## 4. Discussion

To our knowledge, this is the first assessment of the utility of intraoperative neuromonitoring during MIS SI joint fusion using a series of triangular, TPS-coated implants. In spine surgery, iatrogenic nerve injury most commonly occurs directly via mechanical compression, stretching, or laceration or indirectly via ischemia [13]. The extent of neural injury is further dependent on the magnitude, degree, and duration of compression or laceration [14, 15]. Depending on the severity of the initial insult, the resulting injury can range anywhere from recoverable neurapraxia to the irreversible axonotmesis [16]. The current study shows that intraoperative EMG monitoring may be useful in decreasing the potential risk of neural injury utilizing a relatively simple and reproducible technique.

Intraoperative neuromonitoring was first used in 1898 during operations where the facial and trigeminal nerves were at high risk [17]. Advancements in the technique and science of neuromonitoring over the last century have led to its widespread acceptance in several areas of medicine, particularly spinal surgery. One of the earliest applications of IOM in MIS spine surgery was for detecting pedicle wall breaches during pedicle screw placement [18]. Of the various types of IOM, EMG has been highly regarded and well-documented for its ease of use and clinical utility in improving the safety and accuracy of pedicle screw procedures under limited visualization [18–24].

EMG monitoring may likewise prove useful as an adjunct to MIS SI joint fusion surgery as several neural structures, including the cauda equina, L5, S1, and S2 exiting nerves, lie within close proximity to implant trajectory (Figure 1) [25]. Specifically, the trajectory of the most cephalad implant passes in close proximity to the fifth lumbar nerve root [26], while the trajectories of the more caudal implants come close to the first and second sacral foramina, respectively (Figure 1). Not only bony landmarks for placing the implants are often obscured and difficult to visualize on fluoroscopy, but also the anatomy of the sacrum can be highly variable and dysplastic [27]. Safe instrument and implant positioning minimize the potential for neurologic injury [11]. Use of IOM decreases this possibility by providing additional data to assist intraoperative maneuvering during instrumentation and implantation.

Threshold response ranges that indicate a “positive,” or possibly injurious, versus a “negative,” or safe, distance from a nerve must be identified before EMG can be used successfully. Moed et al. studied the correlation of EMG with CT for guide pin placement in the sacrum of a canine animal model [13]. Stimulus-evoked EMG monitoring was used in conjunction with high speed CT imaging to correlate pin tip location within the sacral body to determine a neural proximity EMG response range. A current threshold of 6.3 mA showed the guide pin 1 mm lateral to the sacral canal, while a response ≤5.9 mA resulted in compression or penetration of the L5 nerve root [26]. The authors reported a 0.801 correlation coefficient between recorded thresholds and pin location with respect to the adjacent nerve. Pedicle screw studies in the lower thoracic and lumbosacral spine correlated a threshold range of 8–15 mA as reliable for detecting nerve root proximity [13, 21] and currents of ≤6–10 mA as indicative of possible neural injury [28–30]. A study of 512 lumbar pedicle screw cases determined a threshold response of ≥15 mA to coincide with a 98% confidence that the screw was within the pedicle (as verified via CT), while thresholds between 10 and 15 mA provided an 87% confidence [21].

In the current study, a stimulus of 8 mA was chosen as an upper limit response for initial guide pin placement and 16 mA as the lower limit for implant placement. The lower threshold of 8 mA necessitates a closer proximity to the neural structure to elicit a signal, aiding the surgeon in more accurately determining distance from the nerve. An implant response of 16 mA was chosen based on a combination of literature reports and careful consideration after studying many patients during the SI joint fusion procedure. In the present study, 8 of 111 implants were repositioned following a recording of ≤16 mA. These findings suggest that a neuromonitoring threshold of 16 mA potentially avoided nerve irritation or injury in 7% of device implantations, leading to successful outcomes. These results are similar to those reported in pedicle screw literature, as indicated in the above paragraph [21–23, 28–30].

While intraoperative EMG monitoring adds an element of safety, it is important to note that it is not 100% sensitive and specific. In the current cohort, EMG sensitivity (true positive) was 80% and specificity (true negative) was 97%. The reported average rate of false-negative EMG responses during lumbar surgery is 23% [24]. In a separate study, 2 out of 32 patients were found to develop nerve root irritation in the absence of irregular EMG activity during pedicle screw placement [31]. In the current cohort, two false-negative results were noted. It is worth noting that we never encountered abnormal readings on spontaneous EMG monitoring alone. Only with active stimulation protocols were we able to elicit positive responses. This study emphasizes the need to not rely only on “passive monitoring” or a false sense of security will potentially exist.

In regards to our false positive findings, the patient we brought back to the OR had a superior and lateral sacral deficiency, which is not uncommon. This resulted in an unusually wide SI joint superiorly that was traversed by the implants, especially the superior implant. Our hypothesis is that even though the implants in this case were safely positioned as confirmed by CT, the superior edge of the implant was effectively uncovered for a variable distance by the lack of a bony “roof” due to the dysplasia and, therefore, not as well insulated. This could have contributed to the lower threshold readings.

Such situations demonstrate the technical limitations of neuromonitoring. Specifically, it is imperative that surgeons and neurophysiologists are aware that a negative neuromonitoring reading cannot determine safe implant position with 100% certainty. The benefits of neuromonitoring, when used in conjunction with preoperative CT imaging, fluoroscopy, and meticulous surgical technique, merit its use in MIS SI joint implantation. This surgical adjunctive technique has the potential to optimize positive patient outcomes when implanting medical devices in close proximity to neural structures. However, there is no substitute for experience and sound surgical judgment in each specific case.

Limitations of this study include small sample size, single surgeon experience, and absence of a control group. The small patient size was reflective of the number of patients available in the private practice office. All patients included in this study were followed postoperatively for a minimum of 3 months. The benefits of evaluating patients from a single center include a consistent diagnostic and therapeutic approach. Hopefully, in the near future, other surgeons will add to this body of knowledge and help validate the outcomes of this limited study.

## 5. Conclusions

This study provides insight into the potential benefit of IOM for MIS SI joint fusion. Specifically, results suggest that a stimulation threshold of ≤16 mA may indicate a potentially hazardous implant placement. In addition, the use of an 8 mA search mode technique for initial guide pin placement and final implant position allows the surgeon to determine proximity to adjacent neural structures. Given the potential of these thresholds to decrease the risk of iatrogenic nerve injury, IOM may be a useful adjunct to MIS SI joint fusion surgery.
